# Maternal hypercholesterolemia would increase the incidence of embryo aneuploidy in couples with recurrent implantation failure

**DOI:** 10.1186/s40001-023-01492-x

**Published:** 2023-11-21

**Authors:** Yang Liu, Tianxiang Ni, Qing Zhao, Weiran Cui, Xiangxin Lan, Tingting Zhou, Qian Zhang, Junhao Yan

**Affiliations:** 1 Center for Reproductive Medicine, Shandong University, Jinan, 250012 Shandong China; 2 Key Laboratory of Reproductive Endocrinology of Ministry of Education, Shandong University, Jinan, 250012 Shandong China; 3 Shandong Key Laboratory of Reproductive Medicine, Jinan, 250012 Shandong China; 4 Shandong Provincial Clinical Research Center for Reproductive Health, Jinan, 250012 Shandong China; 5 Shandong Technology Innovation Center for Reproductive Health, Jinan, 250012 Shandong China; 6 National Research Center for Assisted Reproductive Technology and Reproductive Genetics, Shandong University, Jinan, 250012 Shandong China

**Keywords:** Blood lipid, Unexplained repeated implantation failure (uRIF), Preimplantation genetic testing for aneuploidy (PGT-a), Aneuploid rate, Cumulative live birth, Pregnancy outcomes

## Abstract

**Background:**

The association of dyslipidemia with embryo development and pregnancy outcomes is largely unknown, especially in unexplained recurrent implantation failure (uRIF) patients. Here, this study aimed to explore the impact of abnormal blood lipid levels on embryo genetic status and pregnancy outcomes after preimplantation genetic testing for aneuploidy (PGT-A) from a clinical perspective.

**Methods:**

This study retrospectively analyzed 502 patients diagnosed as uRIF. They were divided into four groups according to the levels of cholesterol and triglyceride: nonhyperlipidemia group (NonH group), simple hypercholesterolemia group (SHC group), simple hypertriglyceridemia group (SHC group) and mixed hyperlipidemia group (MixH group). At the same time, patients were divided into non-low HDL-C group and low HDL-C group according to their HDL-C level. The outcomes of embryos genetic testing and pregnancy outcomes after PGT-A was analyzed between groups. Binary logistic regression and/or generalized estimating equation (GEE) model were conducted to investigate the association of different types of dyslipidemia with embryonic aneuploidy rate and cumulative live-birth rate.

**Results:**

474 women who met the inclusion criteria were divided into four groups: NonH group (*N* = 349), SHC group (*N* = 55), SHT group (*N* = 52) and MixH group (*N* = 18). Compared with the NonH group, SHC group had a significantly increased rate of embryo aneuploidy [48.3% vs. 36.7%, *P* = 0.006; adjusted OR (95% confidence interval) = 1.52(1.04–2.22), *P* = 0.029], as well as a reduced number of good-quality embryos on day 5 or 6 [3.00 ± 2.29 vs. 3.74 ± 2.77, *P* = 0.033]. The SHC group showed a tendency of a lower cumulative live birth rate (47.0% vs. 40.0%), a lower incidence of good birth outcome (37.2% vs. 34.5%) and a higher risk of clinical pregnancy loss (11.1% vs. 17.9%), but did not reach statistical significance (*P* > 0.05). The incidences of obstetric or neonatal complications and other adverse events were similar in the four groups. Whether patients have low HDL-C did not differ in pregnancy outcomes.

**Conclusions:**

We found that uRIF women with hypercholesterolemia had an increased proportion of aneuploid embryos and a reduced proportion of high-quality embryos, while different types of hyperlipidemia had no correlation with cumulative live birth rate as well as pregnancy and neonatal outcomes.

**Supplementary Information:**

The online version contains supplementary material available at 10.1186/s40001-023-01492-x.

## Introduction

In recent years, more and more infertile couples have had the chance to deliver babies after treatment with vitro fertilization and embryo transfer [10]. However, 50–60% of couples are still unable to achieve a clinical pregnancy owing to implantation failures [32]. Recurrent implantation failure (RIF) is defined as implantation failures occurring after three or more embryo transfer cycles or after the transfer of four to six embryos at the cleavage stage with high scores or three or more blastocysts with high scores [14]. The etiology of RIF is complex and primarily based on the quality of gametes or embryos and their development potential, the endometrial microenvironment, autoimmune function, prethrombotic state, and other factors [6, 30]. After excluding patients with RIF with the abovementioned common etiologies, a subgroup of patients with RIF whose causes are unknown, defined as unexplained RIF (uRIF), remains. uRIF poses a significant challenge to the advancement of assisted reproductive technologies. Studies have shown that embryo aneuploidy is the major cause of miscarriage or implantation failure [18, 19]. Preimplantation genetic testing for aneuploidy (PGT-A) using next-generation sequencing (NGS) may enhance embryo selection and improve pregnancy outcomes [23]. Thus PGT-A combined with NGS has become one of the main therapeutic methods for couples experiencing uRIF.

Over the past 30 years, the blood cholesterol levels of the Chinese population have gradually increased and the prevalence of dyslipidemia in this population has also grown dramatically [40]. According to a 2012 Chinese national survey report, the prevalence of hypercholesterolemia, hypertriglyceridemia, and low high-density lipoprotein (HDL) cholesterol was 4.9%, 13.1%, and 33.9%, respectively. Moreover, the overall prevalence of dyslipidemia among Chinese adults was as high as 40.40% in 2012, significantly higher than that in 2002. There is growing evidence linking abnormal lipid metabolism to the pathogenesis of various diseases, including cancer and diabetes [24]. Lipid metabolism may be involved in the modulation of sex hormone levels [5, 17, 27]. Dyslipidemia may affect oocyte quality and female fertility, leading to reproductive failure through the induction of oxidative stress [38]. Serum-free cholesterol concentration in women can impact the timing of pregnancy [28] and body fat percentage has been identified as a key parameter determining the success of assisted reproductive technologies [2]. However, the association between the dysregulation of lipid metabolism and RIF remains largely unknown and no relevant study has assessed the impact of different types of dyslipidemia on the development of embryos and their genetic status or on the pregnancy outcomes of patients with RIF.

The bioinformatics analysis performed in our previous study revealed lipid metabolism dysregulation in the endometrium/decidua of patients with RIF, which may be associated with abnormal endometrial receptivity and aberrant immune infiltration [21]. It remains unclear whether abnormal lipid metabolism affects pregnancy outcomes by impairing embryonic development or interfering with the maternal uterine environment. It is crucial to understand how lipid metabolism affects human reproductive function to create tailored medicines that will aid in the efficient diagnosis and treatment of women with RIF [8].

Therefore, taking a clinical perspective, this retrospective study aimed to treat couples experiencing uRIF with PGT-A and further explore the effects of abnormal blood lipid levels on the genetic status of embryos and pregnancy outcomes after transferring euploid embryos.

## Materials and methods

### Patients

We followed the STROBE reporting guidelines specific to our study type. Abnormal blood lipid levels usually refer to elevated serum cholesterol and/or triglyceride levels, commonly known as hyperlipidemia. Dyslipidemia encompasses various lipid disorders, including low HDL-C syndrome. We collected anonymized data from couples experiencing uRIF who underwent their first PGT-A cycle at the Hospital for Reproductive Medicine affiliated with Shandong University between January 2017 and December 2021. RIF was diagnosed based on the 2018 Chinese expert consensus [14]: implantation failures after three or more transfer cycles of good-quality embryos or after transfers of four to six embryos at the cleavage stage with high scores or three or more blastocysts with high scores. uRIF means that no clear causes of implantation failure were found in these patients. The exclusion criteria were as follows: patients with known uterine abnormalities, such as Müllerian duct anomalies, an untreated uterine septum, submucous myoma of the uterus, adenomyosis or endometriosis, endometrial hyperplasia, intrauterine adhesions, or uterine scarring, chromosomal karyotype abnormalities in one of the couples with RIF; polycystic ovary syndrome; and endocrine disorders such as diabetes, immune diseases, coagulation abnormalities, the use of donated oocytes or sperm for pregnancy, or pregnancy contraindications.

The eligible women were divided into nonhyperlipidemia and hyperlipidemia groups based on their serum total cholesterol (TC) levels (≥ 5.2 mmol/L) and/or total triglyceride (TG) levels (≥ 1.7 mmol/L). Further subgroups were created as follows: non–hyperlipidemia group (NonH group; TC < 5.2 mmol/L and TG < 1.7 mmol/L), simple hypercholesterolemia group (SHC group; TC ≥ 5.2 mmol/L and TG < 1.7 mmol/L), simple hypertriglyceridemia group (SHT group; TC < 5.2 mmol/L and TG ≥ 1.7 mmol/L), and mixed hyperlipidemia group (MixH group; TC ≥ 5.2 mmol/L and TG ≥ 1.7 mmol/L). In addition, based on their HDL-C levels, the patients were divided into a non-low HDL-C group (HDL-C ≥ 1.0 mmol/L) and low HDL-C group (HDL-C < 1.0 mmol/L).

This study was approved by the ethics committee of the Hospital for Reproductive Medicine affiliated to Shandong University. Consent from women whose records were included in this study was not available because they were anonymous in our data retrieval system.

### Procedures

Different protocols for controlled ovarian hyperstimulation, such as the long, short, and antagonist protocols, were implemented based on the female ovarian reserve and previous ovarian responses. In the long protocol, 0.05–0.1 mg/d gonadotropin-releasing hormone agonist (GnRH-a) was administered during the midluteal phase of the previous cycle and gonadotropin (Gn) was administered once satisfactory pituitary desensitization had been achieved. In the short protocol, 0.05–0.1 mg/d GnRH-a was administered on the second or third day of the cycle and Gn was administered 2 days later until human chorionic gonadotropin (hCG). In the antagonist protocol, Gn was administered on the third day of the menstrual cycle, and GnRH-a was administered when the diameter of the dominant follicle reached 1.2–1.4 cm. hCG, GnRH-a, or a combination of both was used to trigger final oocyte maturation when the average diameter of at least two follicles reached ≥ 18 mm. Oocyte retrieval was performed 34–36 h later under the guidance of vaginal ultrasonography.

Intracytoplasmic sperm injection was employed in all IVF treatments, with all embryos cultured until the blastocyst stage. Good-quality blastocysts on day 5 or 6 of the embryo culture were selected using the Gardner blastocyst grading system [12], which are based on blastocyst expansion, inner cell quality, and trophoblastic ectodermal development, and subjected to biopsy. Blastocyst biopsy and NGS were performed following the methods reported by Yan et al. [36] and euploid embryos were selected for subsequent transfer.

Single frozen embryo transfers were performed following at least two menstrual cycles after oocyte retrieval. The endometrial preparation regimens included the natural ovulation cycle, ovulation induction cycle, and programmed cycle, as reported previously [13]. Luteal phase support involved dydrogesterone and vaginal progesterone gel administered on the endometrial transformation day and continued until 12 weeks of gestation. Serum hCG levels were measured 2 weeks after transfer to confirm conception. If conception had occurred, transvaginal ultrasonography was performed 3 weeks later to confirm clinical pregnancy, defined by the presence of an intrauterine gestational sac. Transvaginal ultrasonography was repeated at 11 weeks of gestation to confirm ongoing pregnancy. A follow-up was conducted to collect data on all live births or pregnancy terminations, extended until December 2022. All pregnancy and neonatal outcomes were recorded in detail.

### Outcomes

The primary outcome was the embryo aneuploidy rate. PGT-A results were classified into four groups: balanced euploidy, aneuploidy, chromosome mosaic, and questionable outcomes. The secondary outcomes included the cumulative live birth rate after single oocyte retrieval in the first PGT-A cycle as well as the cumulative biochemical, clinical, and ongoing pregnancies; cumulative rates of biochemical and clinical pregnancy loss; birth weight; good birth outcomes [16, 26], pregnancy duration; the number of embryo transfers required to achieve live births; and the cumulative incidence of maternal and neonatal complications. A biochemical pregnancy is characterized by a serum hCG level of at least 25 mU/ml at 14 days after embryo transfer. Clinical pregnancy was confirmed by the presence of an intrauterine gestational sac, as observed using transvaginal ultrasound at 5 weeks after embryo transfer. Pregnancies exceeding 12 weeks were classified as ongoing pregnancies. *Live birth* refers to the delivery of a viable infant at ≥ 28 weeks of gestation. The cumulative live birth rate was calculated by dividing the number of women who delivered a live baby by the total number of women in that group.

### Statistical analysis

The sample size was initially calculated based on the difference in embryo aneuploidy rates. According to our primary hypothesis, we initially planned to test a 10% difference in the embryo aneuploidy rate between the nonhyperlipidemia and hyperlipidemia groups. Considering our preliminarily calculated 35% rate of embryo aneuploidy tested by NGS in the nonhyperlipidemia group, at least 373 blastocysts should be included in each group to detect a 10% absolute elevation in the embryo aneuploidy rate, with 80% power and a 5% two-sided error rate. Normally distributed continuous characteristics are reported as means (± SD) and were compared using independent samples *t*-tests, whereas non-normally distributed continuous characteristics are reported as medians (interquartile ranges) and were compared using the Mann–Whitney *U* test. Categorical variables are reported as frequencies (percentages) and were compared using the Chi-square test. The embryo aneuploidy rate and pregnancy outcomes were compared between the NonH group and the SHC, SHG, or MixH groups separately and totally. Women who were lost to follow-up were considered as not having had a live birth. Binary logistic regression analysis and/or a generalized estimating equation model were conducted to adjust for potential confounding factors and to investigate the association of the different types of dyslipidemia with the embryonic aneuploidy rate and cumulative live birth rate. The potential confounders included age, BMI, and antral follicle count in both ovaries. A two-sided *p*-value of < 0.05 was considered statistically significant. All of the analyses were performed using SPSS software (version 26).

## Results

### Patient and baseline characteristics

**Fig. 1:**
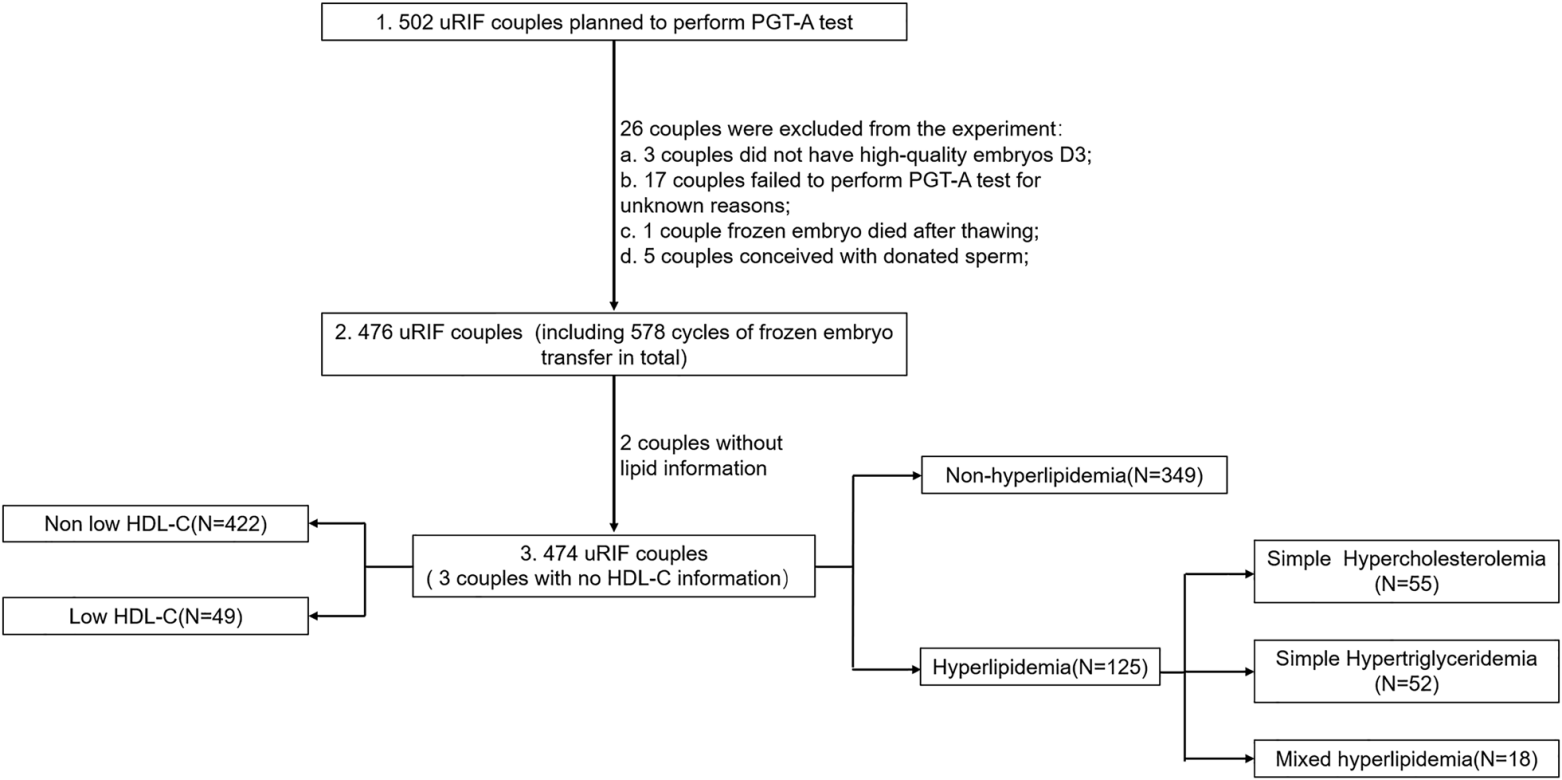
Flow diagram. uRIF, unexplained repeated planting failure; PGT-A, preimplantation genetic testing for aneuploidy

Initially, a total of 502 couples experiencing uRIF were screened (Additional file 1: Table S1). Among these couples, 26 did not undergo PGT-A treatment because they had no good-quality embryos available or had used donated sperm, among other unknown reasons; two other couples had no lipid information available and were thus excluded from the study. Finally, a total of 474 women were included in this study (Fig. 1) and categorized into the nonhyperlipidemia group (*N* = 349; 1142 blastocysts) and hyperlipidemia group (*N* = 125; 376 blastocysts) which can be subdivided into three groups (SHC group [*N* = 55] with 149 blastocysts; SHT group [*N* = 52] with 163 blastocysts; and MixH group [*N* = 18] with 64 blastocysts).

Compared with the NonH group, the SHT group had a higher body mass index (BMI) (25.37 ± 3.87 vs. 23.10 ± 3.03; *p* = 0.000) and a slightly lower serum follicle-stimulating hormone level (6.40 ± 2.04 vs. 7.34 ± 2.67; *p* = 0.016). The other baseline characteristics were comparable in the four groups (Table 1).

**Table 1:** Characteristics of the patients at baseline

Characteristics	NonH (*N* = 349)	SHC (*N* = 55)	P_a_	SHT (*N* = 52)	P_b_	MixH (*N* = 18)	P_c_
Age							
< 38	258/349(73.9%)	37/55(67.3%)	0.302	35/52(67.3%)	0.316	13/18(72.2%)	1.000
≥ 38	91/349(26.1%)	18/55(32.7%)	0.302	17/52(32.7%)	0.316	5/18(27.8%)	1.000
BMI^1^	(23.10 ± 3.03)	(23.91 ± 2.90)	0.067	(25.37 ± 3.87)	0.000	(24.59 ± 2.45)	0.042
Fertility history							
Duration of attempt to conceive—yr	(4.80 ± 3.30)	(4.92 ± 3.56)	0.814	(4.89 ± 3.12)	0.853	(5.00 ± 3.25)	0.806
Previous conception—no./total no. (%)	223/349(63.9%)	34/55(61.8%)	0.766	35/52(67.3%)	0.632	11/18(61.1%)	0.811
Previous miscarriage—no./total no. (%)	195/349(55.9%)	31/55(56.4%)	0.946	31/52(59.6%)	0.612	10/18(55.6%)	0.979
Previous live birth—no./total no. (%)	77/349(22.1%)	12/55(21.8%)	0.968	13/52(25.0%)	0.636	4/18(22.2%)	1.000
Ultrasonographic findings							
Antral follicle count in both ovaries	(14.01 ± 6.93)	(13.38 ± 7.74)	0.542	(17.35 ± 8.84)	0.002	(15.44 ± 12.35)	0.413
Endometrial thickness—mm	(0.78 ± 0.24)	(0.75 ± 0.21)	0.355	(0.79 ± 0.26)	0.749	(0.79 ± 0.22)	0.846
Laboratory testing^2^							
Follicle-stimulating hormone—IU/liter	(7.34 ± 2.67)	(7.49 ± 2.67)	0.687	(6.40 ± 2.04)	0.016	(6.85 ± 2.46)	0.447
Luteinizing hormone—IU/liter	(5.65 ± 4.94)	(4.77 ± 2.27)	0.198	(5.65 ± 4.94)	0.897	(4.61 ± 3.07)	0.380
Estradiol—pg/ml	(82.42 ± 188.95)	(93.18 ± 202.75)	0.698	(103.03 ± 275.14)	0.493	(111.87 ± 261.15)	0.528
Total testosterone—ng/dl	(22.69 ± 14.26)	(25.42 ± 23.09)	0.232	(25.14 ± 18.93)	0.374	(17.11 ± 10.26)	0.102
Prolactin—ng/ml	(18.60 ± 16.51)	(17.71 ± 8.35)	0.694	(16.25 ± 8.52)	0.314	(15.40 ± 9.59)	0.415
TSH—uIU/ml	(2.27 ± 1.12)	(2.27 ± 2.11)	0.999	(2.19 ± 0.83)	0.636	(2.43 ± 0.89)	0.532

NonH, nonhyperlipidemia; SHC, simple hypercholesterolemia; SHT, simple hypertriglyceridemia; MixH, mixed hyperlipidemia

P_a_: NonH group VS SHC group; P_b_: NonH group VS SHT group; P_c_: NonH group VS MixH group

^1^ The body mass index is the weight in kilograms divided by the square of the height in meters

^2^ The baseline steroid hormones were measured at the early follicular phase, mostly on day 1 to 3 of the menstrual cycle

Data were missing regarding TSH in 6 women, PRL in 3 women, TO in 2 women and endometrial thickness in 1 woman

### Outcomes of embryo culturing and genetic testing

No significant difference was observed in the embryo aneuploidy rates between the nonhyperlipidemia and hyperlipidemia groups (36.7% vs. 40.4%; *p* = 0.195). Given that hypercholesterolemia and hypertriglyceridemia may have different impacts on oocyte and embryo development, we focused on assessing the specific variations within the four subgroups.

**Fig. 2:**
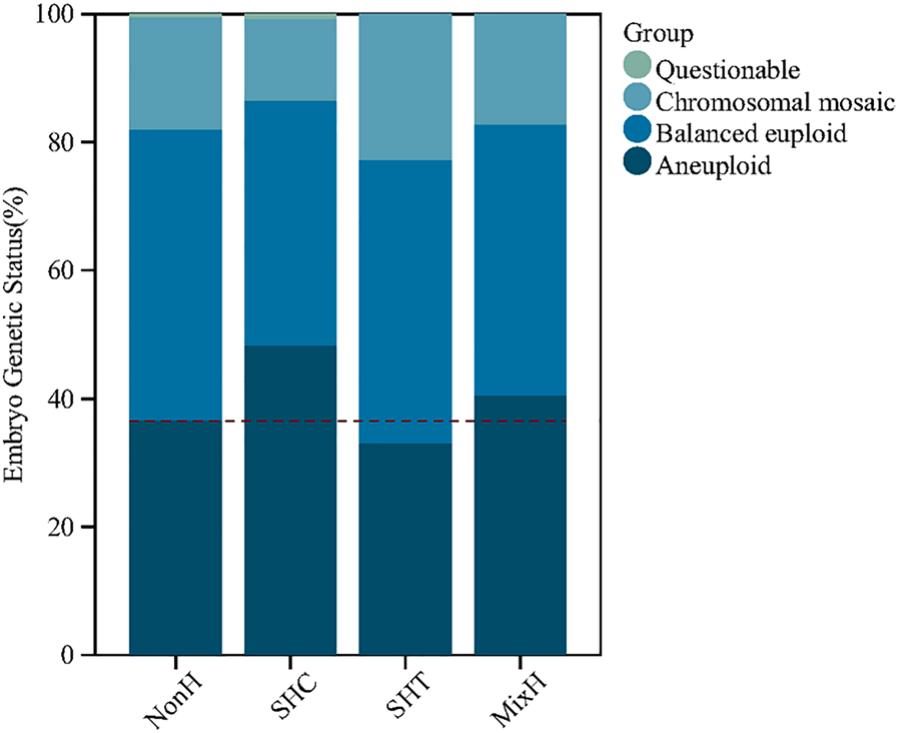
Embryo genetics status. NonH, nonhyperlipidemia; SHC, simple hypercholesterolemia; SHT, simple hypertriglyceridemia; MixH, mixed hyperlipidemia

The results of controlled ovarian hyperstimulation, embryo development, and genetic testing are presented in Table 2. We observed a significantly increased percentage of embryo aneuploidy in the SHC group (48.3% vs. 36.7%; *p* = 0.006) (Fig. 2). In contrast, the percentage of euploidy decreased, but the difference was not statistically significant (38.3% vs. 45.3%; *p* = 0.105). The number of good-quality embryos obtained on day 5 or 6 was smaller in the SHC group than in the NonH group (3.00 ± 2.29 vs. 3.74 ± 2.77; *p* = 0.033). No significant difference was noted in the genetic status of embryos between the NonH and SHT groups or the MixH group. The proportion of women with no euploid embryos for transfer did not differ significantly between each case group and the NonH group.

**Table 2:** Outcomes of controlled ovarian hyperstimulation and embryos

Characteristics^1^	NonH (*N* = 349)	SHC (*N* = 55)	P_a_	SHT (*N* = 52)	P_b_	MixH (*N* = 18)	P_c_
No. of days of ovarian stimulation	(9.68 ± 2.15)	(9.67 ± 1.79)	0.991	(9.79 ± 1.68)	0.718	(9.78 ± 2.18)	0.845
Gonadotropin dosage—IU	(2060.19 ± 879.60)	(2132.27 ± 849.80)	0.571	(2107.21 ± 775.92)	0.715	(2080.56 ± 754.13)	0.923
Estradiol level on hCG trigger day—pg/ml	(3250.41 ± 1920.45)	(3033.78 ± 2101.70)	0.443	(3114.60 ± 2572.00)	0.651	(3277.39 ± 2267.59)	0.954
Endometrial thickness on hCG trigger day—mm	(1.00 ± 0.19)	(0.99 ± 0.18)	0.776	(1.06 ± 0.18)	0.026	(0.98 ± 0.19)	0.735
No. of oocytes retrieved	(10.53 ± 5.94)	(9.80 ± 5.33)	0.389	(10.92 ± 5.25)	0.654	(12.00 ± 10.61)	0.568
No. of good-quality embryos on day 5 or 6	(3.74 ± 2.77)	(3.00 ± 2.29)	0.033	(3.62 ± 2.83)	0.754	(4.11 ± 3.07)	0.587
Result on preimplantation genetic testing—no./total no. (%)							
Balanced euploid	517/1142(45.3%)	57/149(38.3%)	0.105	72/163(44.2%)	0.792	27/64(42.2%)	0.629
Aneuploid	419/1142(36.7%)	72/149(48.3%)	0.006	54/163(33.1%)	0.376	26/64(40.6%)	0.526
Chromosomal mosaic	201/1142(17.6%)	19/149(12.8%)	0.139	37/163(22.7%)	0.115	11/64(17.2%)	0.933
Questionable	5/1142(0.4%)	1/149(0.7%)	0.522	0	1.000	0	1.000
Number of couples having no euploid embryos-no./total no. (%)	107/349(30.7%)	21/55(38.2%)	0.265	15/52(28.8%)	0.791	5/18(27.8%)	0.796

NonH, nonhyperlipidemia; SHC, simple hypercholesterolemia; SHT, simple hypertriglyceridemia; MixH, mixed hyperlipidemia

P_a_: NonH group VS SHC group; P_b_: NonH group VS SHT group; P_c_: NonH group VS MixH group

^1^ The term hCG denotes human chorionic gonadotropin

Data were missing regarding estradiol level on hCG trigger day in 1 woman

### Pregnancy outcomes and the incidence of pregnancy and neonatal complications

The cumulative live birth rates were 47.0% and 44.0% in the nonhyperlipidemia and hyperlipidemia groups, respectively (*p* = 0.565). As shown in Table 3, the cumulative live birth rates and other pregnancy outcomes following the transfer of euploid embryos were also similar in the SHC, SHG, and MixH groups compared with those in the NonH group (*p* > 0.05). However, the SHC group showed a tendency toward a lower cumulative live birth rate (47.0% vs. 40.0%), a lower incidence of good birth outcomes (37.2% vs. 34.5%), and a higher risk of clinical pregnancy loss (11.1% vs. 17.9%), although the differences were not statistically significant. The average number of embryos transferred that resulted in live births was 1.27 ± 0.59 in the nonhyperlipidemia group and 1.09 ± 0.29 in the SHC group (*p* = 0.023). In terms of pregnancy and neonatal complications, the incidence of gestational hypertension, diabetes, and other obstetric or perinatal complications was similar in the four groups (Table 4). Gestational hypertension occurred more frequently in the SHC (12.0%) and SHG (14.3%) groups than in the NonH group (6.0%), but the differences were not statistically significant.

**Table 3:** Cumulative pregnancy outcomes among different groups

Characteristics	NonH (*N* = 349)	SHC (*N* = 55)	P_a_	SHT (*N* = 52)	P_b_	MixH (*N* = 18)	P_c_
Cumulative biochemical pregnancy—no. (%)	199/349(57.0%)	28/55(50.9%)	0.396	28/52(53.8%)	0.667	12/18(66.7%)	0.419
Cumulative clinical pregnancy—no. (%)	183/349(52.4%)	25/55(45.5%)	0.336	28/52(53.8%)	0.849	11/18(61.1%)	0.472
Cumulative ongoing pregnancy—no. (%)	167/349(47.9%)	22/55(40.0%)	0.278	24/52(46.2%)	0.819	10/18(55.6%)	0.524
Cumulative live-birth rate—no. (%)	164/349(47.0%)	22/55(40.0%)	0.334	23/52(44.2%)	0.710	10/18(55.6%)	0.478
Birth weight^1^							
Singleton							
No. of observations	162	22		22		10	
Mean weight—g	(3265.46 ± 568.98)	(3096.82 ± 588.63)	0.196	(3352.86 ± 612.76)	0.512	(3135.00 ± 496.12)	0.480
Twin							
No. of observations	4	0		2		0	
Mean weight—g	2950.00 ± 353.55	0	–	2795.00 ± 1162.63	0.630	0	–
Cumulative pregnancy loss—no./total no. (%)							
Biochemical	21/199(10.6%)	4/28(14.3%)	0.788	2/28(7.1%)	0.822	1/12(8.3%)	1.000
Clinical	22/199(11.1%)	5/28(17.9%)	0.466	5/28(17.9%)	0.466	1/12(8.3%)	1.000
First trimester	17/199(8.5%)	4/28(14.3%)	0.526	3/28(10.7%)	0.981	1/12(8.3%)	1.000
Second trimester	5/199(2.5%)	1/28(3.6%)	0.550	2/28(7.1%)	0.209	0	1.000
Good birth outcome^2^—no. (%)	130/349(37.2%)	19/55(34.5%)	0.699	19/52(36.5%)	0.921	8/18(44.4%)	0.539
Features of live births							
Duration of pregnancy—day	(270.49 ± 14.86)	(267.73 ± 19.29)	0.431	(268.70 ± 12.29)	0.580	(268.10 ± 13.07)	0.619
No. of embryos transferred	(1.27 ± 0.59)	(1.09 ± 0.29)	0.023	(1.13 ± 0.46)	0.183	(1.40 ± 0.52)	0.511
No. of embryo-transfer procedures	(1.27 ± 0.58)	(1.09 ± 0.29)	0.026	(1.13 ± 0.46)	0.201	(1.40 ± 0.52)	0.482

NonH, nonhyperlipidemia; SHC, simple hypercholesterolemia; SHT, simple hypertriglyceridemia; MixH, mixed hyperlipidemia

P_a_: NonH group VS SHC group; P_b_: NonH group VS SHT group; P_c_: NonH group VS MixH group

^1^ Data were missing regarding singleton birth weight in 1 woman and twin birth weight in 1 woman

^2^ A good birth outcome was defined as a live birth at 37 weeks or more of gestation, with a birth weight between 2500 and 4000 g and without a major congenital anomaly

**Table 4:** The incidence of pregnancy and neonatal complications

Characteristics	NonH (*N* = 349)	SHC (*N* = 55)	P_a_	SHT (*N* = 52)	P_b_	MixH (*N* = 18)	P_c_
Maternal							
Gestational diabetes mellitus*	31/183(16.9%)	5/25(20.0%)	0.922	5/28(17.9%)	1.000	1/11(9.1%)	0.793
Preeclampsia or eclampsia*	5/183(2.7%)	1/25(4.0%)	0.541	0	1.000	0	1.000
Gestational hypertension*	11/183(6.0%)	3/25(12.0%)	0.487	4/28(14.3%)	0.233	0	1.000
Preterm delivery*	22/183(12.0%)	2/25(8.0%)	0.797	2/28(7.1%)	0.662	2/11(18.2%)	0.896
Placenta previa*	3/183(%)	0	1.000	0	1.000	0	1.000
Postpartum hemorrhage†	1/164(%)	0	1.000	0	1.000	0	1.000
Fetal, after 12 wk through neonatal period							
Congenital anomaly‡	3/166(1.8%)	0	1.000	2/24(8.3%)	0.121	1/10(10.0%)	0.210
Low birth weight‡^1^	12/166(7.2%)	1/22(4.5%)	0.985	3/24(12.5%)	0.624	1/10(10.0%)	0.545
Macrosomia‡^2^	11/166(6.6%)	0	1.000	1/24(4.2%)	0.989	0	1.000

NonH, nonhyperlipidemia; SHC, simple hypercholesterolemia; SHT, simple hypertriglyceridemia; MixH, mixed hyperlipidemia

P_a_: NonH group VS SHC group; P_b_: NonH group VS SHT group; P_c_: NonH group VS MixH group

^*^ Evaluation was performed in all clinical pregnancies

^†^ Evaluation was performed during or after all deliveries

^‡^ Evaluation was performed in all live newborns

^1^ Low birth weight was defined as a value of less than 2500 g

^2^ Macrosomia was defined as a birth weight of more than 4000 g

The associations between hyperlipidemia and the rates of embryo aneuploidy as well as cumulative live births are shown in Table 5 and Table 6. The generalized estimating equation model showed that simple hypercholesterolemia displayed a significant positive association with the embryo aneuploidy rate after adjusting for the effects of age, BMI, and antral follicle counts in both ovaries (crude OR [95% CI]: 1.68 [1.12–2.52], *p* = 0.013; adjusted OR [95% CI]: 1.52[1.04–2.22], *p* = 0.029). In addition, the logistic regression results showed no correlation between the different types of hyperlipidemia and the cumulative live birth rates (crude OR [95% CI]: 0.75[0.42–1.34], *p* = 0.335; adjusted OR [95% CI]: 0.85[0.46–1.57], *p* = 0.599).

**Table 5:** The associations between hypercholesterolemia and embryo aneuploidy

Variables	Crude OR (95%CI)	*P*	Adjusted OR (95%CI)	*P*
Nonhyperlipidemia group	Ref.	–	Ref.	–
Simple HyperTCemia group	1.68(1.12–2.52)	0.013	1.52(1.04–2.22)	0.029
Simple HyperTGemia group	0.86(0.57–1.28)	0.445	0.77(0.52–1.14)	0.193
Mixed hyperlipidemia group	1.12(0.59–2.15)	0.729	1.00(0.47–2.15)	0.999
Age (< 38)	Ref.	–	Ref.	–
Age (≥ 38)	0.23(0.17–0.32)	0.000	0.25(0.18–0.35)	0.000
BMI	1.02(0.98–1.07)	0.306	1.01(0.97–1.06)	0.586
Antral follicle counts in both ovaries	0.97(0.95–0.98)	0.000	0.99(0.97–1.00)	0.141

**Table 6:** The associations between hypercholesterolemia and cumulative live-birth rate

Variables	Crude OR (95%CI)	*P*	Adjusted OR (95%CI)	*P*
Nonhyperlipidemia group	Ref.	–	Ref.	–
Simple HyperTCemia group	0.75(0.42–1.34)	0.335	0.85(0.46–1.57)	0.599
Simple HyperTGemia group	0.90(0.50–1.61)	0.710	0.94(0.49–1.82)	0.855
Mixed hyperlipidemia group	1.41(0.54–3.66)	0.480	1.56(0.55–4.38)	0.403
Age (< 38)	Ref.	–	Ref.	–
Age (≥ 38)	0.18(0.11–0.29)	0.000	0.22(0.13–0.36)	0.000
BMI	0.96(0.91–1.02)	0.172	0.97(0.91–1.03)	0.302
Antral follicle counts in both ovaries	1.06(1.03–1.09)	0.000	1.04(1.01–1.06)	0.016

### Results for the genetic status of embryos and pregnancy outcomes in patients with low HDL-C syndrome

The patients were simultaneously divided into the non–low HDL-C group (*N* = 422) and low HDL-C group (*N* = 49) based on their HDL levels. The baseline characteristics were similar between the two groups (Additional file 2: Table S2). In addition, no statistically significant differences were noted between the two groups in the rates of embryo euploidy, aneuploidy, and mosaicism (Additional file 2: Table S3); pregnancy outcomes (Additional file 2: Table S4); and maternal and newborn complications (Additional file 2: Table S5).

## Discussion

This study represents the first attempt to assess the impact of varying blood lipid levels on pregnancy outcomes in a population experiencing RIF. In this retrospective study of 474 patients experiencing uRIF, we found that women with hypercholesterolemia exhibited an increased proportion of aneuploid embryos and a reduced proportion of high-quality embryos; in contrast, the different types of hyperlipidemia were not associated with the cumulative live birth rates and pregnancy and neonatal outcomes.

As an essential component of various biological membranes in living organisms, cholesterol plays a pivotal role in multiple biological processes, including cell proliferation and division. Cholesterol has been found to have implications for female reproduction in various species [31]. First, cholesterol governs membrane fluidity; therefore, all proliferating cells require substantial amounts of cholesterol for membrane synthesis [33, 34]. Furthermore, cholesterol serves as an indispensable substrate for steroid synthesis in ovarian follicular cells and is widely considered essential for female fertility [29]. A recent study provided evidence suggesting that maintaining oocyte cholesterol homeostasis is relevant for ensuring the developmental potential of eggs [1]. The cholesterol content within oocytes appears to modulate processes such as maturation, fertilization, activation, and embryo development [3, 35]. Evidence from human in vitro fertilization (IVF) studies suggests that an abnormal maternal serum lipid profile is associated with poorer oocyte quality, compromised ovarian function, and impaired embryo development, all of which cause a potential reduction in fecundity [22]. Furthermore, research has shown that obese women, who often display abnormal serum lipid levels, produce a smaller proportion of good-quality embryos on day 5 and exhibit abnormal expression of genes related to oocyte quality, including *PGR* and *PTX3* [25]. These findings corroborate our observations regarding the association between dyslipidemia and embryo quality. Yesilaltay et al. [35] found that excessive cholesterol exposure could lead mouse eggs to behave as if they had already been fertilized, thereby disrupting the normal synchronization between fertilization and meiosis completion, resulting in dysfunctional eggs. This may be one of the reasons for the increased aneuploidy rate of embryos in women with elevated cholesterol levels, although further research is necessary to confirm this hypothesis.

Embryo quality and endometrial receptivity are the main factors affecting embryo implantation [6, 30]. PGT-A involves selecting euploid embryos for implantation after in vitro fertilization to effectively reduce the risk of an unfavorable pregnancy. Analyzing pregnancy outcomes in patients experiencing RIF who undergo PGT-A treatment can help distinguish the impact of embryos from that of the maternal endometrium, avoiding potential interference from aneuploid embryos as a hybrid factor affecting the conclusions. In addition, the cumulative live birth rate after oocyte retrieval is considered the most crucial patient-centered outcome measure [9, 36]. We found no significant differences in the cumulative live birth rates after transferring euploid embryos between the normal and hyperlipidemia groups. However, we observed that women with hypercholesterolemia exhibited a slightly lower cumulative live birth rate and a slightly higher risk of clinical pregnancy loss; however, these differences were not statistically significant, probably because of the small sample size in the hypercholesterolemia group. Horn et al. [15] reported that the incidence of hypercholesterolemia was higher in women who experienced early miscarriages (< 12 weeks) than in those who had a single live birth. They also showed that hypercholesterolemia was associated with late miscarriages (12–19 weeks). In addition, the clinical parameters and morphological characteristics of the endometrium have been found to be altered in women with abnormal lipid metabolism [20]. Reports have suggested that obesity, which is often accompanied by hypercholesterolemia, negatively impacts endometrial receptivity by delaying the implantation window [4]. Therefore, hypercholesterolemia may increase the risk of pregnancy loss by affecting endometrial receptivity, although further investigations through a clinical study with larger sample sizes and mechanistic studies are warranted to explore the underlying association between hypercholesterolemia and endometrial receptivity.

Emerging evidence suggests that hyperlipidemia is associated with a high incidence of maternal pregnancy complications. Research has reported that the increase in TC levels over time is closely related to the occurrence of diabetes during pregnancy, whereas the increases in TG and low-density lipoprotein cholesterol levels over time are closely related to diabetes and cholestasis during pregnancy [39]. Moreover, oxidative stress caused by disturbances in lipid status may play a role in the onset of preeclampsia in high-risk pregnancies [7]. Our findings indicate that women with hypercholesterolemia and hypertriglyceridemia exhibit an increased risk of gestational hypertension, although this increase may not be statistically significant.

One of the strengths of this study is that we investigated the effects of different types of hyperlipidemia on reproductive outcomes by scientifically grouping blood lipid levels. Second, we included a population experiencing uRIF undergoing PGT-A treatment as the subject, which helped us to distinguish the separate effects of hyperlipidemia on the genetic status of embryos and the maternal endometrium. To the best of our knowledge, this is the first time that such an approach has been employed. However, our research also has some limitations. First, this was a retrospective cohort study with inherent biases. For example, the diversity of the controlled ovarian hyperstimulation protocols may represent different ovarian responses and population heterogeneity, which could have a confounding impact on reproductive outcomes. In addition, the sample size was small, especially in the SHC and SHG groups, making it infeasible to perform subgroup analyses, such as those based on age. Finally, the definition of RIF used in the study was based on the consensus of Chinese experts [14] and, currently, more researchers are beginning to apply the definition criteria newly proposed by ESHRE in 2023 [11]. Thus, the results may not be generalizable to women diagnosed using other criteria.

In conclusion, we found that hypercholesterolemia, as opposed to hypertriglyceridemia, increased the incidence of embryo aneuploidy and reduced the number of good-quality embryos. However, no association was observed between hypercholesterolemia and the cumulative pregnancy outcomes or maternal and neonatal complications. A better understanding of the roles and mechanisms of lipid molecules in regulating the reproductive process will provide valuable insights for developing more effective interventions to address implantation failure [37]. Our findings underscore the negative impacts of dyslipidemia on reproductive outcomes, particularly during oogenesis and embryo development. This suggests that women with hypercholesterolemia should consider taking measures before pregnancy. Further prospective cohort studies are necessary to validate our findings and investigate the underlying mechanisms associated with these observations.

### Supplementary Information

**Additional file 1: Table S1.** Population information.

**Additional file 2: Table S2.** Characteristics of the patients at baseline. **Table S3.** Outcomes of controlled ovarian hyperstimulation. **Table S4.** Cumulative live-birth rate and secondary outcomes. **Table S5.** Adverse events

## Data Availability

Readers can obtain relevant data information through supplementary documents.
