# Supplementary material for: Maternal hypercholesterolemia would increase the incidence of embryo aneuploidy in couples with recurrent implantation failure
Source: Eur J Med Res. 2023 Nov 21;28:534. doi: 10.1186/s40001-023-01492-x (PMC10662148; doi:10.1186/s40001-023-01492-x)
Supplement: Supplementary file 2 — Additional file 2: Table S2. Characteristics of the patients at baseline. Table S3. Outcomes of controlled ovarian hyperstimulation. Table S4. Cumulative live-birth rate and secondary outcomes. Table S5. Adverse events [file 40001_2023_1492_MOESM2_ESM.docx]

Additional TableS2.Characterisics of the patients at baseline.

| Characteristics | Non-low HDL-Cemia(N=422) | Low HDL-Cemia(N=49) | Pvalue |
| --- | --- | --- | --- |
| Age(≥38) | 117/422(27.7%) | 14/49(28.6%) | 0.900 |
| BMI^1^ | (23.21±2.89) | (26.07±4.37) | 0.000 |
| Fertility history |  |  |  |
| Duration of attempt to conceive — yr | (4.91±3.36) | (4.38±2.70) | 0.294 |
| Previous conception — no./total no. (%) | 266/422（63.0%） | 36/49（73.5%） | 0.149 |
| Previous miscarriage — no./total no. (%) | 233/422（55.2%） | 33/49（67.3%） | 0.105 |
| Previous live birth — no./total no. (%) | 94/422（22.3%） | 12/49（24.5%） | 0.725 |
| Ultrasonographic findings |  |  |  |
| Antral follicle count in both ovaries | (13.99±7.10) | (17.49±10.51) | 0.029 |
| Endometrial thickness — mm | (0.78±0.24) | (0.78±0.23) | 0.868 |
| Laboratory testing^2^ |  |  |  |
| Follicle-stimulating hormone — IU/liter | (7.32±2.69) | (6.53±1.70) | 0.005 |
| Luteinizing hormone — IU/liter | (5.57±4.72) | (5.21±2.98) | 0.602 |
| Estradiol — pg/ml | (82.08±182.87) | (132.77±338.48) | 0.307 |
| Total testosterone — ng/dl | (22.65±15.31) | (25.17±19.65) | 0.390 |
| Prolactin — ng/ml | (18.32±15.41) | (16.62±9.01) | 0.448 |
| TSH —uIU/ml | (2.27±1.26) | (2.28±0.94) | 0.992 |

^1^ The body-mass index is the weight in kilograms divided by the square of the height in meters.

^2^ The baseline steroid hormones were measured at the early follicular phase, mostly on day 1 to 3 of the menstrual cycle.

Data were missing regarding TSH in 6 women, PRL in 3 women, TO in 2 women and Endometrial thickness in 1 woman.

Additional TableS3.Outcomes of controlled ovarian hyperstimulation

| Characteristics^1^ | Non-low HDL-Cemia(N=422) | Low HDL-Cemia(N=49) | Pvalue |
| --- | --- | --- | --- |
| No. of days of ovarian stimulation | (9.64±2.06) | (10.14±2.01) | 0.108 |
| Gonadotropin dose — IU | (2049.34±857.35) | (2314.03±862.25) | 0.041 |
| Estradiol level on hCG trigger day — pg/ml | (3254.74±2070.47) | (2913.49±1654.89) | 0.266 |
| Endometrial thickness on hCG trigger day — mm | (1.00±0.19) | (0.99±0.19) | 0.763 |
| No. of oocytes retrieved | (10.38±5.67) | (12.27±8.48) | 0.134 |
| No. of good-quality embryos on day 5 or 6 | (3.64±2.70) | (3.98±3.10) | 0.409 |
| Result on preimplantation genetic testing — no./total no. (%) |  |  |  |
| Balanced euploid | 598/1351(44.3%) | 74/163(45.4%) | 0.783 |
| Aneuploid | 515/1351(38.1%) | 55/163(33.7%) | 0.276 |
| Chromosomal mosaic | 232/1351(17.2%) | 34/163(20.9%) | 0.243 |
| Questionable | 6/1351(0.4%) | 0 | 1.000 |
| Absence of normal embryo | 130/422(30.8%) | 16/49(32.7%) | 0.791 |

| ^1^ The term hCG denotes human chorionic gonadotropin.  Data were missing regarding estradiol level on hCG trigger day in 1 woman. |
| --- |

Additional TableS4. Cumulative live-birth rate and secondary outcomes

| Characteristics | Non-low HDL-Cemia(N=422) | Low HDL-Cemia(N=49) | Pvalue |
| --- | --- | --- | --- |
| Cumulative biochemical pregnancy — no. (%) | 240/422(56.9%) | 27/49(55.1%) | 0.813 |
| Cumulative clinical pregnancy — no. (%) | 222/422(52.6%) | 25/49(51.0%) | 0.833 |
| Cumulative ongoing pregnancy — no. (%) | 200/422(47.4%) | 23/49(46.9%) | 0.952 |
| Cumulative live-birth rate — no. (%) | 197/422(46.7%) | 22/49(43.1%) | 0.632 |
| Birth weight^1^ |  |  |  |
| Singleton |  |  |  |
| No. of observations | 194 | 22 |  |
| Mean weight — g | (3247.81±579.56) | (3277.14±509.33) | 0.824 |
| Twin |  |  |  |
| No. of observations | 6 | 0 |  |
| Mean weight — g | 2872.50±241.85 | 0 | - |
| Cumulative pregnancy loss — no./total no. (%) |  |  |  |
| Biochemical | 26/240(10.8%) | 2/27(7.4%) | 0.826 |
| Clinical | 28/240(11.7%) | 5/27(18.5%) | 0.473 |
| First trimester | 22/240(9.2%) | 3/27(11.1%) | 1.000 |
| Second trimester | 6/240(2.5%) | 2/27(7.4%) | 0.189 |
| Good birth outcome^2^ — no. (%) | 157/422(37.2%) | 18/49(36.7%) | 0.949 |
| Features of live births |  |  |  |
| Duration of pregnancy — day | (2s69.75±15.31) | (271.45±11.72) | 0.613 |
| No. of embryos transferred | (1.23±0.53) | (1.41±0.73) | 0.273 |
| No. of embryo-transfer procedures | (1.22±0.52) | (1.41±0.73) | 0.260 |

^1^ Data were missing regarding singleton birth weight in 1 woman and twin birth weight in 1 woman.

^2^ A good birth outcome was defined as a live birth at 37 weeks or more of gestation, with a birth weight between 2500 and 4000 g and without a major congenital anomaly.

Additional TableS5. Adverse events

| Characteristics | Non-low HDL-Cemia(N=422) | Low HDL-Cemia(N=49) | Pvalue |
| --- | --- | --- | --- |
| Maternal |  |  |  |
| Gestational diabetes mellitus* | 38/222(17.1%) | 4/25(16.0%) | 1.000 |
| Preeclampsia or eclampsia* | 6/222(2.7%) | 0 | 1.000 |
| Gestational hypertension* | 15/222(6.8%) | 4/25(16.0%) | 0.212 |
| Preterm delivery* | 26/222(11.7%) | 2/25(8.0%) | 0.824 |
| Placenta previa* | 3/222(1.4%) | 0 | 1.000 |
| Postpartum hemorrhage† | 1/198(0.5%) | 0 | 1.000 |
| Fetal, after 12 wk through neonatal period |  |  |  |
| Congenital anomaly‡ | 5/200(2.5%) | 1/22(4.5%) | 0.469 |
| Low birth weight‡^1^ | 16/200(8.0%) | 1/22(4.5%) | 0.876 |
| Macrosomia‡^2^ | 11/200(5.5%) | 1/22(4.5%) | 1.000 |

* Evaluation was performed in all clinical pregnancies.

† Evaluation was performed during or after all deliveries.

‡ Evaluation was performed in all live newborns.

^1^ Low birth weight was defined as a value of less than 2500 g.

^2^ Macrosomia was defined as a birth weight of more than 4000 g.
